# Coronary Computed Tomographic Angiography at 80 kVp and Knowledge-Based Iterative Model Reconstruction Is Non-Inferior to that at 100 kVp with Iterative Reconstruction

**DOI:** 10.1371/journal.pone.0163410

**Published:** 2016-09-22

**Authors:** Joohee Lee, Chul Hwan Park, Chi Suk Oh, Kyunghwa Han, Tae Hoon Kim

**Affiliations:** 1 Department of Radiology and Research Institute of Radiological Science, Gangnam Severance Hospital, Yonsei University College of Medicine, Seoul, Republic of Korea; 2 Department of Radiology, Research Institute of Radiological Science, Severance Hospital, Yonsei University College of Medicine, Seoul, Republic of Korea; City University London, UNITED KINGDOM

## Abstract

The aims of this study were to compare the image noise and quality of coronary computed tomographic angiography (CCTA) at 80 kVp with knowledge-based iterative model reconstruction (IMR) to those of CCTA at 100 kVp with hybrid iterative reconstruction (IR), and to evaluate the feasibility of a low-dose radiation protocol with IMR. Thirty subjects who underwent prospective electrocardiogram-gating CCTA at 80 kVp, 150 mAs, and IMR (Group A), and 30 subjects with 100 kVp, 150 mAs, and hybrid IR (Group B) were retrospectively enrolled after sample-size calculation. A BMI of less than 25 kg/m^2^ was required for inclusion. The attenuation value and image noise of CCTA were measured and the signal-to-noise ratio (SNR) and contrast-to-noise ratio (CNR) were calculated at the proximal right coronary artery and left main coronary artery. The image noise was analyzed using a non-inferiority test. The CCTA images were qualitatively evaluated using a four-point scale. The radiation dose was significantly lower in Group A than Group B (0.69 ± 0.08 mSv vs. 1.39 ± 0.15 mSv, *p* < 0.001). The attenuation values were higher in Group A than Group B (*p* < 0.001). The SNR and CNR in Group A were higher than those of Group B. The image noise of Group A was non-inferior to that of Group B. Qualitative image quality of Group A was better than that of Group B (3.6 vs. 3.4, *p* = 0.017). CCTA at 80 kVp with IMR could reduce the radiation dose by about 50%, with non-inferior image noise and image quality than those of CCTA at 100 kVp with hybrid IR.

## Introduction

Coronary computed tomographic angiography (CCTA) has been considered a reliable imaging modality for detecting and ruling out coronary artery disease (CAD) [1], but there are still concerns regarding radiation exposure [2]. Currently, there is interest in obtaining qualified diagnostic images while reducing radiation exposure to as low as reasonably achievable. This is a problem associated with cardiac computed tomography (CT) that has not been solved [3].

Recently, iterative reconstruction (IR) has been widely used for CCTA image reconstruction; it is advantageous in that it can reduce the noise that is caused by a decrease in spatial resolution in traditional filtered back-projection (FBP) algorithms [4–6]. By using this technique, it was possible to reduce the dose through decreasing the tube potential to 100 kVp through IR. Conventional FBP reconstruction algorithms have been shown to result in substantial increases in noise with the use of a 100 kVp instead of a 120 kVp protocol [4]. However, it has been difficult to further reduce the tube voltage to 80 kVp with IR as a consequence of increased noise and degradation of image quality [7–9].

Knowledge-based iterative model reconstruction (IMR) using a fully iterative algorithm has been applied in the clinic to help reduce noise while maintaining image quality compared to IR [10,11]. We hypothesized that combining the low-dose CCTA protocol at 80 kVp with IMR could overcome the above limitations and provide acceptable image quality. The objective of the study was to assess the non-inferiority of the CCTA protocol at 80 kVp with IMR compared to CCTA at 100 kVp using hybrid IR by performing quantitative and qualitative analyses of image noise and quality.

## Material and Methods

### Study population

This study was designed as a retrospective observational study. Thirty subjects (14 men and 16 women; mean age: 53.0 ± 9.5 years) who underwent prospective ECG-gating CCTA at 80 kVp, 150 mAs, and IMR (Level 1; Group A) were retrospectively enrolled in the study between August and November of 2014. An additional 30 subjects (18 men and 12 women; mean age: 55.2 ± 7.6 years) who underwent prospective ECG-gating CCTA at 100 kVp, 150 mAs, with hybrid IR (iDose^4^-Level 5; Group B) were also enrolled between April and July of 2014. The inclusion criteria were a body mass index (BMI) of less than 25 kg/m^2^, coronary calcium load of less than 400 Agatston units [9], and age greater than 20 years. Medical records and CT images were reviewed retrospectively. The exclusion criteria were a heart rate of greater than 65 bpm before CT, known arrhythmia, previous allergic reaction to iodinated contrast media, renal insufficiency (serum creatinine greater than 150 μmol/L), hemodynamic instability, and congestive heart failure. Our institutional review board (Gangnam Severance Hospital IRB) approved this study and written informed consent was waived due to the retrospective nature of this study. Patient records and information were anonymized and de-identified prior to analysis

### Imaging protocol

All CCTA procedures were performed on a 64-slice CT scanner (Ingenuity Core 128, Philips Healthcare, Cleveland, Ohio, U.S.A.). Data acquisition was performed in the craniocaudal direction during a single breath-hold at the end-inspiratory pause. The scanning range encompassed the heart from the level of the carina to the diaphragm. Prospective ECG-triggering was used in all cases with the step-and-shoot axial scanning technique. If the heart rate before CCTA was greater than 65 beats per minute (bpm), a β-blocker (40–80 mg propranolol hydrochloride; Pranol, Dae Woong, Seoul, Korea) was administered orally 1 h before examination. No additional β-blockers were intravenously administered at the time of examination. Subjects who had arrhythmia or heart rates above 65 bpm immediately before or during the examination underwent retrospective CCTA using an ECG-modulation protocol and were excluded from the study.

The scanning parameters were as follows: step-and-shoot axial scanning, 80 kVp or 100 kVp tube voltage, 150 mAs tube current, 64 × 0.625 mm detector collimation with dynamic z-focal spot imaging, 400-ms gantry rotation time, 4-cm table feed per rotation, and the center of the imaging window was set at 70–80% of the R‒R interval. Ioversol with an iodine concentration of 350 mg/mL (Optiray 350; Tyco Healthcare, Kantata, Canada) was administered intravenously through an antecubital 18-gauge catheter at a rate of 0.75 mL/kg for 15 s, followed by 50 mL of normal saline, using a power injector (Dual Shot; Nemoto Kyorindo, Tokyo, Japan). The start time of data acquisition was determined using a real-time bolus tracking technique and scans began 7 s after attaining a trigger threshold of 130 HU in the proximal descending aorta. The breath-hold maneuver was successfully performed in all scans. The ECG signal was recorded simultaneously during each study. The effective radiation dose for CCTA was calculated by multiplying the dose‒length product (DLP) with a conversion coefficient of 0.014 mSv/(mGy × cm) [12].

### CT image reconstruction

CT images were reconstructed using IMR (IMR-level 1; Philips Healthcare, Cleveland, Ohio, U.S.A.) in Group A and hybrid IR (iDose^4^-level 5) in Group B. The images were then transferred to a picture archiving and communication system (PACS; Centricity 2.0, GE Medical Systems, Mount Prospect, IL, U.S.A.). The reconstruction parameters were the following: 0.9-mm slice thickness, 0.45-mm increment, 512 × 512 pixel image matrix, XCC kernel, and a 15–23 cm field of view. Post-processing and reconstruction were performed for qualitative evaluation with multi-planar and curved-planar reformatted images, using commercially available software (Aquarius Workstation V3.6, TeraRecon, San Mateo, CA, U.S.A.).

### Quantitative analysis

All images were reviewed and interpreted on PACS workstations. The vascular attenuation values for the two groups of axial CT images were measured using a round region of interest larger than 1.5 cm^2^ at the ascending aorta, at the proximal right coronary artery (RCA), and at the left main coronary artery (LM). Image noise was evaluated in CCTA based on one standard deviation of the attenuation value at the ascending aorta. The signal-to-noise ratio (SNR) was calculated as vascular attenuation/image noise. The contrast-to-noise ratio (CNR) was calculated as [(attenuation of vessel)—(attenuation of the adjacent perivascular fat)]/image noise. These parameters were then compared between the two groups.

### Qualitative analysis

Two radiologists who had more than 10 years of experience interpreting cardiac imaging independently assessed CCTA image quality. The readers were blinded to all patient identity, clinical information, and reconstruction method. Coronary segments were subdivided according to the 18-segment model of the Society of Cardiovascular Computed Tomography guidelines [13]. Segments with a diameter of at least 1.5 mm at the origin were included. The window level to interpret image of CCTA used at the mean of the Hounsfield unit (HU) values with a width of 800, and a level of 300 as a starting point, with readjustments for body habitus, extent of calcification, and contrast intensity [13]. The image quality of the coronary segments was assessed using a four-point grading scale as follows: Grade 1 (poor/non-diagnostic), severe image degradation or discontinuation of vessel contour that prevented vessel lumen evaluation; Grade 2 (adequate), moderate image degradation that impeded vessel lumen evaluation; Grade 3 (good), minor image degradation that did not affect vessel lumen evaluation; and Grade 4 (excellent), no image degradation.

### Statistical analysis

Sample size calculation was based on a margin of non-inferiority for image noise of 5.0 using preliminary image noise measurements that were obtained for 10 subjects, who were not included in the final study population. We found that at least 30 subjects were required in each group in order to obtain a power of 90% and a two-sided α-level of 0.05 to demonstrate the non-inferiority of the 80 kVp with IMR protocol.

Continuous variables were expressed as the mean ± standard deviation and categorical variables were expressed as frequencies or percentages. Shapiro‒Wilk tests were used to evaluate the distribution of the data. Independent two-sample *t*-tests were performed to assess differences in demographic data, including age, height, weight, BMI, and average heart rate between the two groups. Fisher’s exact tests were used to evaluate differences in sex between the two groups. Independent two-sample *t*-tests were used to determine the statistical significance of differences in CT attenuation, SNR, CNR, and radiation dose between the two groups. A two-sided 95% confidence interval (CI) was calculated for the difference in image noise between the 80 kVp with IMR and 100 kVp with hybrid IR protocols to test for non-inferiority in image noise [14]. The non-inferiority margin for the difference in image noise between the two groups was set as 5.0.

The mean image quality scores between the two groups were compared using a linear mixed model considering the interaction effect. Inter-reader agreement for qualitative image quality of CCTAs was measured using the linearly weighted κ statistic. κ values were defined as follows: 0, no agreement; 0–0.2, poor agreement; 0.21–0.40, fair agreement; 0.41–0.60, moderate agreement; 0.61–0.80, substantial agreement; and 0.81–0.99, nearly perfect agreement. A *p*-value less than 0.05 was considered statistically significant. All statistical analyses were performed with commercially available software with the Power Analysis and Sample-Size package (Version 12) and the SPSS 20 Statistical Package for the Social Sciences (Chicago, IL, U.S.A.).

## Results

### Characteristics of the subjects

In this study, we examined a total of 60 subjects (30 in Group A and 30 in Group B). The demographic data for the subjects are summarized in Table 1. The age, height, weight, BMI, and average heart rate did not differ between the two groups (*p* > 0.05). All CCTA procedures were performed without complications.

**Table 1:** Demographic data for the 30 subjects in Group A and Group B.

Characteristics	Group A	Group B	*p*-value
**Number of subjects**	30	30	
**Age (years)**	53.0 ± 9.5	55.2 ± 7.5	0.332
**Men:Women**	14:16	18:12	0.438
**Height (cm)**	164.2 ± 7.6	166.3 ± 7.6	0.288
**Body weight (kg)**	59.7 ± 8.1	62.2 ± 7.8	0.244
**Body mass index (kg/m**^**2**^**)**	22.0 ± 1.6	22.4 ± 1.7	0.407
**Average heart rate (beats/min)**	53.4 ± 3.9	54.1 ± 4.2	0.512
**Effective radiation dose (mSv)**	0.69 ± 0.08	1.39 ± 0.15	<0.001
**CTDIvol (mGy)**	3.49 ± 0.11	7.26 ± 0.13	<0.001
**Z-axis (cm)**	14.15 ± 1.64	13.68 ± 1.55	0.261
**Agaston calcium score**			
** Median**	0	0	0.061
** Interquartile range**	0	7.75	
** Mean ± SD**	2.1 ± 7.8	5.5 ± 10.5	

Data are presented as the mean ± standard deviation.

### Quantitative analysis

**Fig 1:**
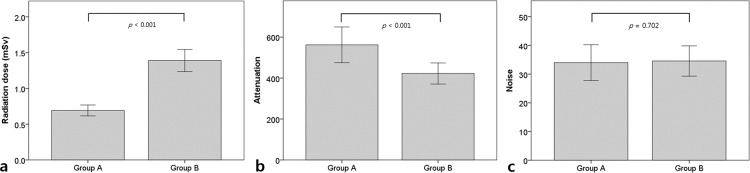
**Comparison of radiation dose, computed tomography attenuation, and image noise between Group A and Group B.** (a) The mean radiation dose in Group A is lower (sub-mSv levels) than that of Group B (*p* < 0.001). (b) The mean computed tomography attenuation is higher in Group A than in Group B (*p* < 0.001). (c) The image noise is not statistically different between Group A and B (*p* = 0.702).

**Fig 2:**
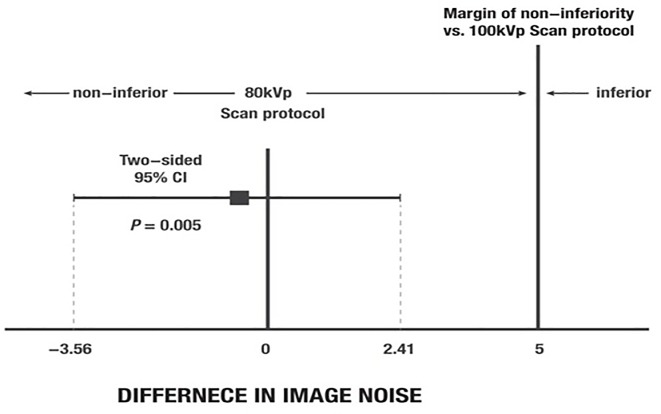
Differences in noise in coronary computed tomographic angiography images at the aortic root between Group A and Group B. The non-inferiority of the image noise with the 80 kVp scan protocol was confirmed, because the upper limit of the two-sided 95% confidence interval (CI) of the mean image noise difference was smaller than the pre-defined non-inferiority margin of 5.0 (mean difference: -0.57, 95% CI: -3.56–2.41).

There was a significant reduction in the mean radiation dose in Group A compared to Group B, which was measured by the estimated effective dose (0.69 ± 0.08 mSv vs. 1.39 ± 0.15 mSv, *p* < 0.001). The mean CT attenuation values of the aortic root in CCTA were higher in Group A than in Group B (546.3 ± 66.8 HU vs. 419.8 ± 49.7 HU, *p* < 0.001; Fig 1). The mean attenuation values at the LM and RCA were also higher in Group A than in Group B (562.4 ± 87.2 HU vs. 429.2 ± 61.2 HU in the LM and 587.7 ± 95.1 HU vs. 438.3 ± 68.1 HU in the RCA, *p* < 0.001). The SNR of the LM and RCA was significantly higher in Group A than in Group B (17.2 ± 4.6 vs. 12.6 ± 2.2 in the LM and 18.0 ± 4.8 vs. 12.9 ± 2.4 in the RCA, *p* < 0.001). The CNR at the LM and the RCA were significantly higher in Group A than in Group B (19.8 ± 5.1 vs. 15.4 ± 2.7 in the LM and 20.9 ± 5.4 vs. 15.9 ± 2.7 in the RCA, *p* < 0.001) (Table 2, Fig 1). The non-inferiority of the image noise in Group A was demonstrated since the upper limit of the two-sided 95% CI of the mean image noise difference was smaller than the pre-defined non-inferiority margin of 5.0 (mean difference: -0.57, 0.95% CI: -3.56–2.41) (Fig 2).

**Table 2:** Quantitative analysis of coronary computed tomographic angiography image quality in Group A compared to Group B.

	Group A	Group B	*p*-value
**Attenuation of the aortic root**	546.3 ± 66.8	419.8 ± 49.7	< 0.001
**Attenuation of the LM**^**a**^	562.4 ± 87.2	429.2 ± 61.3	< 0.001
**Attenuation of the RCA**^**b**^	587.7 ± 95.1	438.3 ± 68.1	< 0.001
**Noise**	33.9 ± 6.2	34.5 ± 5.3	0.702
**SNR**^**c**^ **of the LM**	17.2 ± 4.6	12.6 ± 2.2	< 0.001
**SNR of the RCA**	18.0 ± 4.8	12.9 ± 2.4	< 0.001
**CNR**^**d**^ **of the LM**	19.8 ± 5.1	15.4± 2.7	< 0.001
**CNR of the RCA**	20.9 ± 5.4	15.9 ± 2.7	< 0.001

Data are presented as the mean ± standard deviation

Abbreviations

^a^Left main coronary artery

^b^Right coronary artery

^c^Signal-to-noise ratio

^d^Contrast-to-noise ratio.

### Qualitative analysis and inter-reader agreement

In Group A, a total of 376 segments with diameters greater than 1.5 mm were evaluated, and all 376 segments were scored as diagnostic (Grade 2–4). There were 267 segments (71.0%) that were considered excellent (Grade 4), 87 (23.1%) good (Grade 3), and 22 (5.9%) adequate (Grade 2). No segments were considered non-diagnostic (Grade 1).

**Fig 3:**
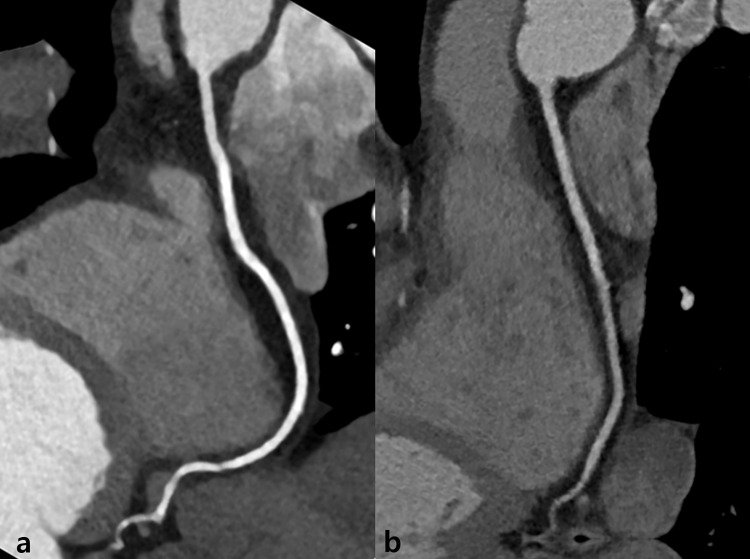
Representative coronary computed tomographic angiography (CCTA) images in Group A and Group B. Curved multi-planar images of the right coronary artery in Group A (a) and Group B (b). Note the substantial reduction in noise and the significantly increased vessel attenuation in (a) compared to (b). (a) A 48-year-old woman underwent CCTA using step-and-shoot axial scanning, 80 kVp tube voltage, and 150 mAs tube current for coronary disease screening. She was 160 cm tall and weighed 49 kg (body mass index: 19.1 kg/m^2^). Her mean heart rate during CCTA was 54 bpm, and the effective radiation dose was 0.63 mSv. The mean attenuation of the ascending aorta was 536.1 HU. The image noise on CCTA was 26.7. The overall image quality was 3.8. (b) A 53-year-old woman underwent CCTA using step-and-shoot axial scanning, 100 kVp tube voltage, and 150 mAs tube current for coronary disease screening. She was 166 cm tall and weighed 52 kg (body mass index: 18.9 kg/m^2^). Her mean heart rate during CCTA was 56 bpm, and the effective radiation dose was 1.33 mSv. The mean attenuation of the ascending aorta was 374 HU. The image noise on CCTA was 31.7. The overall image quality was 3.5.

In Group B, a total of 398 segments with diameters greater than 1.5 mm were evaluated. Of these segments, 224 (56.3%) were considered excellent (Grade 4), 134 (33.7%) good (Grade 3), 36 (9.0%) adequate (Grade 2), and 4 (1%) poor (Grade 1, non-diagnostic). In a per-segment analysis, the graded image quality scores for a few segments, which included the right posterior descending artery, middle left anterior descending artery, distal left anterior descending artery, distal left circumflex artery, and ramus intermedius, were higher in Group A than in Group B. In addition, the mean image quality of the total coronary segments of Group A was significantly higher than Group B (*p* = 0.017) (Fig 3). The detailed segmental evaluation results are shown in Table 3. The inter-reader agreement for visual grading was 0.770 (95% CI: 0.687–0.853) for the 80 kVp protocol and 0.845 (95% CI: 0.781–0.909) for the 100 kVp protocol.

**Table 3:** Qualitative analysis of the images produced for Group A versus B.

	Group A (80 kVp)	Group B (100 kVp)	*p*-value
**Image quality:**			
**Per-segment analysis**			
** Mean of 18 segments**	3.65 ± 0.59	3.45 ± 0.70	**0.017**
** pRCA**^**a**^	4 ± 0	3.97 ± 0.18	0.813
** mRCA**^**b**^	3.83 ± 0.38	3.72 ± 0.53	0.480
** dRCA**^**c**^	3.71 ± 0.46	3.69 ± 0.54	0.891
** RPD**^**d**^	3.5 ± 0.67	2.89 ± 0.88	**0.003**
** RPLB**^**e**^	3.23 ± 0.73	3.06 ± 0.8	0.450
** LM**^**f**^	4.00 ± 0.00	3.93 ± 0.25	0.636
** pLAD**^**g**^	3.97 ± 0.18	3.87 ± 0.35	0.478
** mLAD**^**h**^	3.9 ± 0.31	3.6 ± 0.5	**0.033**
** dLAD**^**i**^	3.63 ± 0.49	3.17 ± 0.53	**0.001**
** D1**^**j**^	3.34 ± 0.77	3.27 ± 0.69	0.565
** D2**^**k**^	3.13 ± 0.74	3.17 ± 0.71	0.681
** pLCx**^**l**^	3.93 ± 0.25	3.8 ± 0.48	0.344
** OM1**^**m**^	3.38 ± 0.71	3.23 ± 0.87	0.257
** dLCx**^**n**^	3.25 ± 0.7	2.92 ± 0.69	**0.031**
** OM2**^**o**^	3 ± 0.71	3.20 ± 0.45	0.521
** L-PDA**^**p**^	3 ± 0	2 ± 0	0.261
** RI**^**q**^	3.5 ± 0.55	2.93 ± 0.73	**0.021**
** L-PLB**^**r**^	2.4 ± 0.55	2.67 ± 1.03	0.332
**Image quality:**			
**Per-vessel analysis**			
** RCA**	3.85 ± 0.36	3.80 ± 0.46	0.501
** LAD**	3.83 ± 0.37	3.54 ± 0.54	**< 0.001**
** LCx**	3.60 ± 0.62	3.39 ± 0.73	**0.038**
**No. of segments**			
**No. of segments in each score (%)**	376 (100)	398 (100)	
** Grade 4 (Excellent)**	267 (71.0)	224 (56.3)	
** Grade 3 (Good)**	87 (23.1)	134 (33.7)	
** Grade 2 (Adequate)**	22 (5.9)	36 (9.0)	
** Grade 1 (Poor)**	0 (0)	4 (1)	

Data are presented as the mean ± standard deviation

Abbreviations

^**a**^Proximal right coronary artery

^b^Middle right coronary artery

^c^Distal right coronary artery

^d^Right posterior descending artery

^e^Right postero-lateral branch

^f^Left main coronary artery

^g^Proximal left anterior descending artery

^h^Middle left anterior descending artery

^i^Distal left anterior descending artery

^j^First diagonal branch

^k^Second diagonal branch

^l^Proximal left circumflex artery

^m^First obtuse marginal branch

^n^Distal left circumflex artery

^o^Second obtuse marginal branch

^p^Posterior descending artery from the left circumflex artery

^q^Radmus intermedius

^r^Left postero-lateral branch from the left circumflex artery. Segments with a diameter of at least 1.5 mm at the origin were included.

## Discussion

The objective of this study was to assess the feasibility of a low tube voltage protocol with IMR reconstruction. In comparison to CCTA at 100 kVp with hybrid IR, CCTA at 80 kVp with IMR in non-obese individuals allowed a 50% reduction in the radiation exposure and showed non-inferior image noise and subjective image quality.

Previously, CCTA was introduced as a useful, non-invasive diagnostic imaging modality for the detection of CAD. However, the radiation dose from CCTA was a concern because of the potential stochastic cancer risks associated with medical radiation exposure [2,15]. Therefore, various strategies and scanning protocols that allow reduction in the radiation dose of CCTA have been developed, including lowering the tube voltage, prospective ECG triggering, and tube current modulation [16–19]. Using a decreased tube voltage is one effective way of reducing radiation dose. In comparison to a 100-kVp scan protocol, data acquisition using a tube voltage of 80 kVp was associated with a 47% reduction in radiation exposure [8]. However, lowering the tube voltage resulted in increased noise due to photon starvation [7].

The IR systems for CT use hybrid methods that combined FBP with multifrequency noise removal techniques to help reduce noise uniformly in both the projection, and image spaces in order to improve image quality or to compensate for high noise caused by lower tube current acquisitions using hybrid IR. The newly introduced IMR technique, which relies on statistical and system models and approaches reconstruction as an optimization process, could theoretically result in noise-free images while improving image quality through the iterative minimization of the penalty-based cost function [5,10,20,21].

Studies that evaluated the effectiveness of IMR for CCTA have indicated that IMR improved image quality and reduced image noise compared to other reconstruction methods when using a conventional 120-kVp protocol [10,22,23]. Yuki et al. [24] compared IMR, IR, and FBP at 100 kVp in non-obese patients who underwent cardiac CT and reported that IMR resulted in better image quality with less noise and fewer artifacts. Oda et al. [11] also showed that an IMR algorithm could provide improved qualitative and quantitative image quality compared to IR and FBP in low-dose CCTA when using a 100-kVp protocol and prospective ECG-gated scanning. Stehli at el. [25] have also reported accurate noninvasive diagnosis of coronary artery disease with the use of a model-based IR-reconstructed CCTA. In our previous study, we reported the feasibility of using CCTA with 80 kVp, 200 mAs, prospective ECG gating, and an IMR algorithm to produce images of markedly higher quality (3.6 ± 0.6), compared with the use of IR and FBP (3.1 ± 0.7 for IR; 2.3 ± 0.6 for FBP, *p* < 0.01) [26]. However, to our knowledge, no study has compared the effectiveness of a reduced tube voltage (80 kVp) combined with IMR to the widely used 100 kVp CCTA protocol combined with IR.

In the present study, we used low-dose CCTA of 80 kVp and 150 mAs, and a prospective, ECG-gated scan protocol. In comparison to CCTA at 100 kVp, the 80 kVp protocol of CCTA in non-obese subjects resulted in a nearly 50% reduction of the mean radiation dose to sub-mSv levels. The quantitative image quality obtained with 80 kVp and IMR was better than that obtained with 100 kVp and IR, considering interaction effects (3.65 ± 0.59 vs. 3.45 ± 0.70, *p* = 0.017). In addition, in this study, lowering the tube voltage to 80 kVp with the updated IR algorithm of IMR could provide non-inferior image noise compared to CCTA at 100 kVp using hybrid IR. Thus, the results demonstrated that image noise did not differ significantly when the tube voltage was reduced.

In clinical studies, lowering the tube voltage resulted in higher vascular contrast enhancement when using a similar contrast-dose protocol [27,28]. Iodine attenuation is expected to increase as the tube voltage decreases, because the energy of an X-ray photon approximates the k-absorption edge of iodine, resulting in increased attenuation [29]. In our study, reducing the tube voltage to 80 kVp with the IMR protocol resulted in a more than 30% increase in iodine attenuation compared to the 100-kVp technique, when performing the same iodine contrast media injection protocol. We demonstrated that reduced tube voltage with IMR could enhance the CNR and SNR by increasing iodine attenuation, as well as reducing the radiation dose; therefore, it could provide improved quantitative and qualitative image quality, while achieving non-inferior image noise in comparison to hybrid IR at 100 kVp. Additionally, since the low radiation dose CCTA protocol showed non-inferiority in image noise, as well as high image attenuation using IMR reconstruction, the amount of contrast media administered could be reduced with an appropriate SNR and CNR, thereby reducing the adverse effects of the contrast materials.

### Limitations

There were several limitations in this study. First, the number of subjects that were enrolled retrospectively was too low for the results to be generalizable. However, we analyzed an adequate number of subjects to demonstrate the non-inferiority of the 80 kVp with the IMR protocol. Second, the present study was limited by the lack of randomization of selected subjects and by the potential selection bias. In addition, the two different types of imaging acquisition protocols were not compared in the same patients. However, in this retrospective observational study, the subjects were sequentially enrolled and evaluated based on the inclusion criteria, and there were no differences in demographics between the two groups. Finally, the diagnostic performance in terms of CAD was not assessed in this study; instead, we focused on image quality and noise. However, image quality was assessed qualitatively by two radiologists and it was determined that the image noise was sufficiently low. In addition, no segments were non-diagnostic. In future, we plan to analyze the diagnostic accuracy of low tube voltage prospective CCTA with IMR by including a larger number of subjects.

## Conclusions

In conclusion, CCTA in non-obese subjects at 80 kVp with IMR was associated with a 50% reduction in radiation dose, with non-inferior image noise and quality than CCTA using 100 kVp and hybrid IR.

## Supporting Information

S1 File Attached files are data of quantitative and qualitative image qualities of CCTA. (XLSX)

## Abbreviations

(BMI): Body mass index
(CT): Computed tomography
(CI): Confidence interval
(CNR): Contrast-to-noise ratio
(CAD): Coronary artery disease
(CCTA): Coronary computed tomographic angiography
(DLP): Dose-length product
(ECG): Electrocardiogram
(FBP): Filtered back projection
(IMR): Iterative model reconstruction algorithm
(IR): Iterative reconstruction
(LAD): Left anterior descending artery
(LM): Left main coronary artery
(pRCA): Proximal right coronary artery
(SNR): Signal-to-noise ratio

## References

[1] Miller JM, Rochitte CE, Dewey M, Arbab-Zadeh A, Niinuma H, Gottlieb I, et al Diagnostic performance of coronary angiography by 64-row CT. N Engl J Med. 2008;359: 2324–2336. 10.1056/NEJMoa0806576 19038879

[2] Einstein AJ, Henzlova MJ, Rajagopalan S. Estimating risk of cancer associated with radiation exposure from 64-slice computed tomography coronary angiography. Jama. 2007;298: 317–323. 17635892 10.1001/jama.298.3.317

[3] Leschka S, Stolzmann P, Schmid FT, Scheffel H, Stinn B, Marincek B, et al Low kilovoltage cardiac dual-source CT: attenuation, noise, and radiation dose. Eur Radiol. 2008;18: 1809–1817. 10.1007/s00330-008-0966-1 18392829

[4] Silva AC, Lawder HJ, Hara A, Kujak J, Pavlicek W. Innovations in CT dose reduction strategy: application of the adaptive statistical iterative reconstruction algorithm. AJR Am J Roentgenol. 2010;194: 191–199. 10.2214/AJR.09.2953 20028923

[5] Hou Y, Liu X, Xv S, Guo W, Guo Q. Comparisons of image quality and radiation dose between iterative reconstruction and filtered back projection reconstruction algorithms in 256-MDCT coronary angiography. AJR Am J Roentgenol. 2012;199: 588–594. 10.2214/AJR.11.7557 22915398

[6] Klink T, Obmann V, Heverhagen J, Stork A, Adam G, Begemann P. Reducing CT radiation dose with iterative reconstruction algorithms: the influence of scan and reconstruction parameters on image quality and CTDIvol. Eur J Radiol. 2014;83: 1645–1654. 10.1016/j.ejrad.2014.05.033 25037931

[7] Tang K, Wang L, Li R, Lin J, Zheng X, Cao G. Effect of low tube voltage on image quality, radiation dose, and low-contrast detectability at abdominal multidetector CT: phantom study. J Biomed Biotechnol. 2012;2012: 130169 10.1155/2012/130169 22619490 PMC3347747

[8] LaBounty TM, Leipsic J, Poulter R, Wood D, Johnson M, Srichai MB, et al Coronary CT angiography of patients with a normal body mass index using 80 kVp versus 100 kVp: a prospective, multicenter, multivendor randomized trial. AJR Am J Roentgenol. 2011;197: W860–867. 10.2214/AJR.11.6787 22021533

[9] Oda S, Utsunomiya D, Yuki H, Kai N, Hatemura M, Funama Y, et al Low contrast and radiation dose coronary CT angiography using a 320-row system and a refined contrast injection and timing method. J Cardiovasc Comput Tomogr. 2015;9: 19–27. 10.1016/j.jcct.2014.12.002 25677790

[10] Oda S, Utsunomiya D, Funama Y, Katahira K, Honda K, Tokuyasu S, et al A knowledge-based iterative model reconstruction algorithm: can super-low-dose cardiac CT be applicable in clinical settings? Acad Radiol. 2014;21: 104–110. 10.1016/j.acra.2013.10.002 24331272

[11] Oda S, Weissman G, Vembar M, Weigold WG. Iterative model reconstruction: improved image quality of low-tube-voltage prospective ECG-gated coronary CT angiography images at 256-slice CT. Eur J Radiol. 2014;83: 1408–1415. 10.1016/j.ejrad.2014.04.027 24873832

[12] Jessen KA, Shrimpton PC, Geleijns J, Panzer W, Tosi G. Dosimetry for optimisation of patient protection in computed tomography. Appl Radiat Isot. 1999;50: 165–172. 10028635 10.1016/s0969-8043(98)00024-4

[13] Leipsic J, Abbara S, Achenbach S, Cury R, Earls JP, Mancini GJ, et al SCCT guidelines for the interpretation and reporting of coronary CT angiography: a report of the Society of Cardiovascular Computed Tomography Guidelines Committee. J Cardiovasc Comput Tomogr. 2014;8: 342–358. 10.1016/j.jcct.2014.07.003 25301040

[14] Ahn S, Park SH, Lee KH. How to demonstrate similarity by using noninferiority and equivalence statistical testing in radiology research. Radiology. 2013;267: 328–338. 10.1148/radiol.12120725 23610094

[15] Brenner DJ, Hall EJ. Computed tomography—an increasing source of radiation exposure. N Engl J Med. 2007;357: 2277–2284. 18046031 10.1056/NEJMra072149

[16] Bischoff B, Hein F, Meyer T, Hadamitzky M, Martinoff S, Schomig A, et al Impact of a reduced tube voltage on CT angiography and radiation dose: results of the PROTECTION I study. JACC Cardiovasc Imaging. 2009;2: 940–946. 10.1016/j.jcmg.2009.02.015 19679281

[17] Hausleiter J, Martinoff S, Hadamitzky M, Martuscelli E, Pschierer I, Feuchtner GM, et al Image quality and radiation exposure with a low tube voltage protocol for coronary CT angiography results of the PROTECTION II Trial. JACC Cardiovasc Imaging. 2010;3: 1113–1123. 10.1016/j.jcmg.2010.08.016 21070998

[18] Hausleiter J, Meyer TS, Martuscelli E, Spagnolo P, Yamamoto H, Carrascosa P, et al Image quality and radiation exposure with prospectively ECG-triggered axial scanning for coronary CT angiography: the multicenter, multivendor, randomized PROTECTION-III study. JACC Cardiovasc Imaging. 2012;5: 484–493. 10.1016/j.jcmg.2011.12.017 22595156

[19] Hlaihel C, Boussel L, Cochet H, Roch JA, Coulon P, Walker MJ, et al Dose and image quality comparison between prospectively gated axial and retrospectively gated helical coronary CT angiography. Br J Radiol. 2011;84: 51–57. 10.1259/bjr/13222537 21172966 PMC3473817

[20] Halpern EJ, Gingold EL, White H, Read K. Evaluation of coronary artery image quality with knowledge-based iterative model reconstruction. Acad Radiol. 2014;21: 805–811. 10.1016/j.acra.2014.02.017 24809321

[21] Chang W, Lee JM, Lee K, Yoon JH, Yu MH, Han JK, et al Assessment of a model-based, iterative reconstruction algorithm (MBIR) regarding image quality and dose reduction in liver computed tomography. Invest Radiol. 2013;48: 598–606. 10.1097/RLI.0b013e3182899104 23511193

[22] Scheffel H, Stolzmann P, Schlett CL, Engel LC, Major GP, Karolyi M, et al Coronary artery plaques: cardiac CT with model-based and adaptive-statistical iterative reconstruction technique. Eur J Radiol. 2012;81: e363–369. 10.1016/j.ejrad.2011.11.051 22197733

[23] Fuchs TA, Stehli J, Bull S, Dougoud S, Clerc OF, Herzog BA, et al Coronary computed tomography angiography with model-based iterative reconstruction using a radiation exposure similar to chest X-ray examination. Eur Heart J. 2014;35: 1131–1136. 10.1093/eurheartj/ehu053 24553723 PMC4006092

[24] Yuki H, Utsunomiya D, Funama Y, Tokuyasu S, Namimoto T, Hirai T, et al Value of knowledge-based iterative model reconstruction in low-kV 256-slice coronary CT angiography. J Cardiovasc Comput Tomogr. 2014;8: 115–123. 10.1016/j.jcct.2013.12.010 24661824

[25] Stehli J, Fuchs TA, Bull S, Clerc OF, Possner M, Buechel RR, et al Accuracy of coronary CT angiography using a submillisievert fraction of radiation exposure: comparison with invasive coronary angiography. J Am Coll Cardiol. 2014;64: 772–780. 10.1016/j.jacc.2014.04.079 25145520

[26] Park CH, Lee J, Oh C, Han KH, Kim TH. The feasibility of sub-millisievert coronary CT angiography with low tube voltage, prospective ECG gating, and a knowledge-based iterative model reconstruction algorithm. Int J Cardiovasc Imaging. 2015;31 Suppl 2: 197–203. 10.1007/s10554-015-0795-7 26521066

[27] Huda W, Scalzetti EM, Levin G. Technique factors and image quality as functions of patient weight at abdominal CT. Radiology. 2000;217: 430–435. 11058640 10.1148/radiology.217.2.r00nv35430

[28] Nakayama Y, Awai K, Funama Y, Hatemura M, Imuta M, Nakaura T, et al Abdominal CT with low tube voltage: preliminary observations about radiation dose, contrast enhancement, image quality, and noise. Radiology. 2005;237: 945–951. 16237140 10.1148/radiol.2373041655

[29] Nakayama Y, Awai K, Funama Y, Liu D, Nakaura T, Tamura Y, et al Lower tube voltage reduces contrast material and radiation doses on 16-MDCT aortography. AJR Am J Roentgenol. 2006;187: W490–497. 17056879 10.2214/AJR.05.0471

